# Barosensory vessel mechanics and the vascular sympathetic baroreflex: Impact on blood pressure homeostasis

**DOI:** 10.1113/EP089686

**Published:** 2023-04-09

**Authors:** Guto W. Hughes, Jonathan P. Moore, Rachel N. Lord

**Affiliations:** ^1^ School of Human and Behavioural Sciences Bangor University Bangor UK; ^2^ Centre for Activity, Health and Wellbeing Research Cardiff Metropolitan University Cardiff UK

**Keywords:** baroreflex, barosensory vessel unloading mechanics, healthy ageing, muscle sympathetic nerve activity, sympathetic nervous system

## Abstract

An age‐associated increase in arterial blood pressure is evident for apparently healthy humans. This is frequently attributed to stiffening of the central arteries and a concurrent increase in sympathetic outflow, potentially mediated by a reduced ability of the baroreceptive vessels to distend. This is supported, in part, by a reduced mechanical component of the vascular sympathetic baroreflex (i.e., a reduction in distension for a given pressure). Previous characterization of the mechanical component has assessed only carotid artery distension; however, evidence suggests that both the aortic and carotid baroreflexes are integral to blood pressure regulation. In addition, given that baroreceptors are located in the vessel wall, the change in wall tension, comprising diameter, pressure and vessel wall thickness, and the mechanics of this change might provide a better index of the baroreceptor stimulus than the previous method used to characterize the mechanical component that relies on diameter alone. This brief review summarizes the data using this new method of assessing barosensory vessel mechanics and their influence on the vascular sympathetic baroreflex across the lifespan.

## Abstract (highlights)

- **What is the topic of this review?**
  We review barosensory vessel mechanics and their role in blood pressure regulation across the lifespan.
- **What advances does it highlight?**
  In young normotensive men, aortic unloading mechanics contribute to the resting operating point of the vascular sympathetic baroreflex; however, with advancing age, this contribution is removed. This suggests that barosensory vessel unloading mechanics are not driving the well‐documented age‐related increase in resting muscle sympathetic nerve activity.

## 1 REFLEX CONTROL OF BLOOD PRESSURE

Arterial blood pressure is the controlled variable of the cardiovascular system. Fundamentally, rapid, short‐term reflex pressure regulation is initiated by stretch‐sensitive baroreceptors located in the walls of the aorta and carotid sinus. An increase in blood pressure engages aortic and carotid sinus baroreceptors and activates afferent input to CNS cardiovascular control centres. A drop in blood pressure has the opposite effect on arterial baroreceptors and afferent activity. Notably, baroreceptor reflex control of the circulation is exerted through two distinct efferent pathways: the cardiovagal baroreflex and the vascular sympathetic baroreflex. Each pathway is capable of regulating blood pressure independently, to some extent. The cardiovagal baroreflex mainly controls heart rate and cardiac output, whereas the vascular sympathetic baroreflex primarily controls arteriolar tone and total peripheral resistance. Overall, the integrated response to an increase in mean arterial blood pressure and baroreceptor loading consists of reduced heart rate, cardiac output and total peripheral resistance; effects that restrain the initial increase in blood pressure. On the contrary, the response to a drop in arterial pressure and the resultant baroreceptor unloading is increased heart rate, cardiac output and total peripheral resistance that counteract the fall in blood pressure. Importantly, studies by Ogoh et al. (2002, 2003) demonstrated that reflex‐mediated changes in arterial pressure are evoked initially by a rapid change in heart rate and cardiac output, but over time there is a dramatic shift to the vascular sympathetic component. Ultimately, the baroreceptor reflex represents a powerful, negative feedback control system for buffering large swings in arterial blood pressure, thus maintaining an optimal pressure (the ‘set point’).

## 2 FUNCTIONAL ASSESSMENT OF THE VASCULAR SYMPATHETIC BAROREFLEX

Assessment of vascular sympathetic baroreflex function in humans is achieved using sympathetic microneurography, a technique pioneered in the 1960s, in which a fine tungsten needle inserted into a peripheral nerve is used to record efferent impulse traffic. Traditionally, this technique has been used to study modulation of muscle sympathetic nerve activity (MSNA) by arterial baroreceptors (Vallbo et al., 2004). However, in a recent development, Macefield and colleagues used ultrasound‐guided microneurography to make the first in‐human recordings of efferent activity in the left and right cervical vagus nerves (Ottaviani et al., 2020; Patros et al., 2022). Although limited to efferent activity, this innovative approach might be an important first step to enable microneurographic recording of vagal afferent nerve activity in humans. Even so, owing to the present inability to measure afferent activity in humans directly, much of our core understanding of baroreceptor function comes from animal studies, in which manipulated baroreceptor loading and concurrent afferent activity can be recorded. Animal studies have demonstrated that alterations at barosensory sites affecting arterial baroreceptor input can alter afferent activity and have a sustained, long‐term effect on blood pressure control, with chronic unloading generating neurogenic hypertension secondary to elevated sympathetic outflow (Thrasher, 2002). Although we can only speculate about baroreceptor loading and consequent afferent activity in humans, the concept that baroreceptors might influence resting sympathetic outflow is supported in humans following carotid body tumour removal (Timmers et al., 2003) and by animal models.

Baroreflex function is often quantified by assessing the baroreflex sensitivity, also referred to as baroreflex gain. This is the change in effector response (R–R interval or MSNA) for a given change in distending pressure. Baroreflex sensitivity can be broken down further into the mechanical, neural and integrated components: the mechanical transduction of pressure into change in arterial diameter, the neural transduction of diameter change to effector response, and the integrated effect of pressure changes directly on effector response. The mechanical component is viewed as a non‐invasive surrogate of baroreceptor loading but is not without limitations. Previous assessment of the mechanical component of the arterial baroreflex has quantified only the systolic diameter of the common carotid artery in response to the corresponding pressure (Hunt et al., 2001). However, this approach does not give an indication of the magnitude or time course of baroreceptor loading and unloading, nor does it address mechanical pressure transduction in aortic baroreceptors, which also play an important role in reflex control of sympathetic vasomotor activity. Importantly, the sensitivity of the baroreflex to a given distending pressure differs dependent on whether this occurs during a rise or fall in systemic pressure, with distinct responses evident in both the neural and mechanical components (Studinger et al., 2009).

## 3 THE IMPACT OF HEALTHY AGEING

Even in apparently healthy humans, blood pressure increases with advancing age. To some extent, this can be attributed to structural and functional changes in the circulatory system related to arterial stiffening, particularly aortic stiffening (Mitchell et al., 2004). This increase in arterial stiffening is mirrored by a progressive increase of vascular sympathetic outflow with advancing age, perhaps attributable to reduced ability of the baroreceptive vessels to distend. However, despite this upward trend in both blood pressure and MSNA with age, the sympathetic baroreflex sensitivity is maintained, albeit at a higher operating pressure (Ebert et al., 1992; Monahan, 2007). Although overall integrated sensitivity is maintained, there is some evidence to suggest a reduced mechanical component, secondary to arterial stiffening, that is compensated for by an increased neural component (Studinger et al., 2009). In contrast, the cardiovagal arm of the baroreflex displays not only a similar age‐related resetting of the operating point but also a significant loss in sensitivity (Ebert et al., 1992; Monahan, 2007; Monahan et al., 2001). It has been suggested that the increased neural component of the vascular sympathetic baroreflex might be necessary to offset the age‐associated reduction in cardiovagal baroreflex gain; that is, dependence on vascular sympathetic baroreflex outflow for blood pressure homeostasis increases with advancing age (Jones et al., 2001). It is therefore difficult to determine whether the age‐associated increase in vascular sympathetic activity is caused by a dysregulation of the systems governing their inhibition or reflects an adaptation to maintain the overall responsiveness of autonomic blood pressure regulation as the cardiovagal system degrades (Taylor & Tan, 2014).

## 4 NEW METHOD TO CHARACTERIZE THE MECHANICAL COMPONENT

In our recent study (Lord et al., 2020), we calculated systolic and diastolic wall tension in the aorta and carotid artery and used these as surrogates for baroreceptor loading and unloading. Given that wall tension is determined from diameter, pressure and vessel wall thickness and that baroreceptors are located within the vessel wall, we reasoned that our approach might provide a better index of the baroreceptor stimulus than the previously used method to characterize the mechanical component. This previous method, established by Hunt et al. (2001) for the assessment of the cardiovagal baroreflex, uses the correlation between systolic blood pressure and ultrasound‐derived systolic carotid artery diameter as the index of the mechanical component of integrated cardiovagal baroreflex gain. This method has only been used once in the assessment of the vascular sympathetic baroreflex, by Studinger et al. (2009), who used the correlation between diastolic blood pressure and diastolic carotid artery diameter as their index for the mechanical component of integrated vascular sympathetic baroreflex gain. The use of systolic versus diastolic diameter highlights the opposing blood pressure metric on which integrated gain in the different arms of the baroreflex is based: systolic blood pressure in the cardiovagal arm and diastolic blood pressure in the vascular sympathetic arm. Irrespective of the systolic or diastolic diameter, these assessments are based on a single diameter measure at one point of the cardiac cycle, as opposed to the change in diameter for a given pulse pressure.

Moreover, given that MSNA bursts occur during diastole, we considered the possibility that the magnitude and rate of unloading and the time spent unloaded during diastole might be important in regulating the operating point of the vascular sympathetic baroreflex. Notably, it is suggested that the occurrence of a burst of MSNA is determined via a central gating mechanism, in which the generation and strength of a burst might be dependent on the magnitude and duration of baroreceptor afferent activity (Kienbaum et al., 2001). Hence, the duration of recoil when blood pressure falls during diastole might be an important modulator of the burst gating system, with longer periods for baroreceptor unloading leading to a higher likelihood of burst occurrence (Kienbaum et al., 2001).

Despite both aortic and carotid baroreceptor sites supplying afferent inputs, the vast majority of sympathetic baroreflex research uses the carotid arteries as their measurement site, probably owing to their relative ease of access for imaging and manipulation. However, previous studies have suggested that the aortic baroreflex might have a stronger influence than the carotid baroreflex on blood pressure regulation in young men (Sanders et al., 1989) and that aortic baroreceptors have a higher pressure mechanosensitivity than carotid baroreceptors (Lau et al., 2016). Although studies of the carotid baroreceptors have generated a wealth of information, the lack of focus on the aortic baroreceptors signifies a considerable gap in baroreflex research that we sought to address in our study (Lord et al., 2020).

## 5 THE IMPACT OF BAROSENSORY VESSEL MECHANICS ON THE VASCULAR SYMPATHETIC BAROREFLEX

Interestingly, despite increased arterial stiffness in the middle‐aged cohort, the magnitude of wall tension unloading was similar between young and middle‐aged men in both the carotid and aortic measures. However, there were significant differences in the rate of unloading and the time spent unloaded at both aortic and carotid sites, reflecting the expected age‐related changes in the mechanical properties of the vessels and loss of elastic recoil. A positive relationship between aortic unloading mechanics, but not carotid unloading mechanics, and the MSNA operating point was evident in young men. This relationship suggests not only that aortic unloading mechanics might play an important role in MSNA regulation, but also that the system places a differential importance on aortic baroreceptor activity over the carotid. This mirrors the findings of an older publication, in which the relative influences of the aortic and carotid baroreceptors were assessed (Sanders et al., 1989). Using a combination of vasodilatory drugs, external neck suction and external neck pressure to manipulate receptor activation, the baroreceptors were exposed to disparate stimuli. The results demonstrated that baroreflex function was impaired whenever activation of the aortic baroreceptors, but not carotid baroreceptors, was manipulated, suggesting that activation of the aortic baroreceptors appears to supersede that of the carotid receptors. Interestingly, the relationship between aortic unloading mechanics and the MSNA operating point was not present in middle‐aged men. Age‐related stiffening inherent to the middle‐aged group caused slower responses to dynamic pressure changes and return to diastolic diameter, resulting in a shorter time spent unloaded; this could have reduced the opportunity for a burst of MSNA to occur. However, it is well understood that MSNA increases, rather than decreases, with age, suggesting that any control input from baroreceptor activity evident in younger men might disappear by middle age. The authors suggest that either adaptation of the baroreceptor with age or central neural remodelling might be responsible for the elevated MSNA in the middle‐aged group.

Animal studies show that baroreceptor activity declines following a period of sustained pressure elevation with increased vessel stiffness, which might be related to reduced stretch sensitivity (Andresen et al., 1980) and therefore a higher threshold needed to initiate baroreceptor afferent activity (Chapleau et al., 1989). The possibility that central neural remodelling might also alter sympathetic outflow at rest is conceivable (Chapleau et al., 1995). In addition, Osborn et al. (2005) propose that there is a baroreceptor‐independent CNS set point for sympathetic outflow. However, neither of these mechanisms can be assessed easily in vivo in humans.

## 6 FUTURE RESEARCH DIRECTIONS

The study by Lord et al. (2020) provides a new surrogate for baroreceptor loading, and the key findings from the study are intriguing with respect to baroreceptor control inputs for resting sympathetic outflow. However, the rigour of the study was limited, using only 15 cardiac cycles for the aortic ultrasound data. Collection of continuous, time‐aligned physiological data and ultrasound images would allow for beat‐by‐beat analysis of MSNA burst activity relative to changes in wall tension. Although software for automated tracking of vessel diameter is commercially available, the continual calculation of wall tension is not yet possible but is likely to be achievable soon. Additionally, the increase in the quantity of data provided by a continuous, time‐aligned approach would facilitate study of baroreflex hysteresis, which requires a greater volume of data to generate sensitivity regressions for both rising and falling pressures. Analysis of hysteresis in combination with other new methods in future research could expand the understanding of age‐ and disease‐related compensatory mechanisms in autonomic blood pressure control. In addition to more in‐depth analysis using beat‐by‐beat data, further investigation designed to influence baroreceptor unloading mechanics using manipulations of volume/pressure in barosensory vessels is required to establish the importance of unloading mechanics in determining the level of sympathetic outflow. Future research should also aim to determine the relative contributions of aortic and carotid baroreceptors by the use of neck collar suction/pressure during acute manipulations of volume/pressure. Although both the aortic and carotid arteries stiffen with advancing age, there is some evidence to suggest that the magnitude of stiffening might be higher in the aorta compared with the carotid artery (Paini et al., 2006). A greater magnitude of arterial stiffening would impact on unloading mechanics and, potentially, their role in determining the operating point of the vascular sympathetic baroreflex.

The results from Lord et al. (2020) only provide information relating to male participants. Sex‐related differences in neural control and autonomic regulation of blood pressure are widely reported (Briant et al., 2016; Casey et al., 2011; Hart et al., 2009); therefore, these results might not be generalizable to women. Up to the age of ∼40 years, the vascular sympathetic baroreflex operates around a lower MSNA set point (Narkiewicz et al., 2005), but with similar responsiveness, for women compared with men. Notably, women possess more compliant arteries and display lower mean arterial pressure than men (Doonan et al., 2013; Smulyan et al., 2001), which is related to the lower MSNA set point. Thus, it might be possible that unloading mechanics will represent a similar control input in young females to that observed for males, albeit with a larger magnitude of unloading in the setting of more complaint arteries in women (Ogola et al., 2018). That said, women demonstrate a different relationship between MSNA, total peripheral resistance and cardiac output, suggesting that women might rely on different physiological mechanisms to maintain blood pressure (Hart et al., 2009). Although women experience a similar age‐related increase in MSNA, the rate of increase is more significant than in men (Narkiewicz et al., 2005) and is likely to be mediated by the distinct change in sex hormones, changes in β‐adrenergic responsiveness (Harvey et al., 2014) and/or arterial stiffening (Ogola et al., 2018) following the menopause. It is possible that any control input seen in younger women might not exist in older women and might be mediated by the significant increase in arterial stiffness with advancing age in women compared with men, and consequential disruption to unloading mechanics. This might explain, at least in part, the increase in MSNA. Future studies should focus on determining the impact of sex on baroreceptor control of resting sympathetic outflow.

## 7 CONCLUSION

In our view, the increase in MSNA with healthy ageing might indicate an increased dependence on the vascular sympathetic baroreflex for blood pressure homeostasis. Notably, barosensory vessel mechanics do not appear to influence the outflow of the vascular sympathetic baroreflex in healthy middle‐aged men. In contrast, aortic mechanics are associated with the operating point of the vascular sympathetic baroreflex in young men. This suggests that the elevated basal sympathetic outflow with advancing age is not driven by vascular ageing and stiffening of barosensitive vessel walls. Therefore, central mechanisms that subserve baroreflex resetting are likely to underpin elevated basal vasomotor outflow with ageing. Further exploration of the potential control input of barosensory vessel mechanics and the mechanisms responsible for increased sympathetic outflow with age are fundamental to our understanding of the progression of hypertension and other cardiovascular diseases.

## AUTHOR CONTRIBUTIONS

Guto W. Hughes, Rachel N. Lord and Jonathan P. Moore contributed to the conception or design of the work, interpretation of data for the work and drafting of the work or revising it critically for important intellectual content. All authors approved the final version of the manuscript and agree to be accountable for all aspects of the work in ensuring that questions related to the accuracy or integrity of any part of the work are appropriately investigated and resolved.

## CONFLICT OF INTEREST

None declared.

## FUNDING INFORMATION

None.
